# Supplementary material for: TwoStepCisMR: A Novel Method and R Package for Attenuating Bias in cis-Mendelian Randomization Analyses
Source: Genes (Basel). 2022 Aug 26;13(9):1541. doi: 10.3390/genes13091541 (PMC9498486; doi:10.3390/genes13091541)
Supplement: Supplementary file 1 [file genes-13-01541-s001.zip › genes-1847918-supplementary (1).pdf]

Supplementary Methods

Derivation of the standard errors (SE)

Propagation of error

Under the propagation of error rules

$$SE_{A+B} = \sqrt{SE_A^2 + SE_B^2}$$

and

$$SE_{A*B} = A * B * \sqrt{\left({SE_A}/{B_A}^2 + {SE_B}/{B_B}^2\right)}$$

The estimate we are interested in is:

$$B_{g-o\ (adjsuted)} = B_{g-o\ (crude)} - B_{g-c} * B_{c-o}$$

Where $B_{g\text{-}o\ (adjusted)}$ is the adjusted variant-outcome association, $B_{g\text{-}o\ (crude)}$ is the crude variant-outcome association, $B_{g\text{-}c}$ is the variant-confounder association, and $B_{c\text{-}o}$ is the confounder-outcome association.

It follows that the standard error (SE) from following the rules of propagation of error rules is:

$$EPSE = \sqrt{SE_{g-o\ (curde)}^2 + \left(B_{g-c} * B_{c-o}\right)^2 * \left({SE_{g-c}}/{B_{g-c}}^2 + {SE_{c-o}}/{B_{c-o}}^2\right)}$$

Where EPSE is the propagation of error standard error, $SE_{g\text{-}o\ (crude)}$ is the standard error of the crude variant-outcome association, $B_{g\text{-}c}$ is the variant-confounder association, and $B_{c\text{-}o}$ is the confounder-outcome association, $SE_{g\text{-}c}$ is the standard error of the variant-confounder association, and $SE_{c\text{-}o}$ is the standard error of the confounder-outcome association.

Parametric bootstrap standard error

Let:

$$D_{g-o\ (crude)} \sim N(B_{g-o\ (crude)}, {SE_{g-o\ (crude)}}^2)$$

$$D_{g-c} \sim N(B_{g-c}, {SE_{g-c}}^2)$$

$$D_{c-o} \sim N(B_{c-o}, {SE_{c-o}}^2)$$

Where, $B_{g\text{-}o\ (crude)}$ is the crude variant-outcome association, $SE_{g\text{-}o\ (crude)}$ is the standard error of the crude variant-outcome association, $B_{g\text{-}c}$ is the variant-confounder association, and $B_{c\text{-}o}$ is the confounder-outcome association, $SE_{g\text{-}c}$ is the standard error of the variant-confounder association, and $SE_{c\text{-}o}$ is the standard error of the confounder-outcome association.

Then if we define D’ as:

$$D' = D_{g-o\,(crude)} - (D_{g-c} * D_{c-o})$$

Then bootstrap standard error can then be defined as the standard deviation in $D_{g\text{-}o\,(crude)}$:

$$BSSE = \sqrt{\frac{\sum_i^n \left(\overline{D'} - D'_i\right)^2}{n}}$$

Where BSSE is the bootstrap standard error, and n is the number of iterations used in the bootstrap, and $\overline{D'}$ is the mean value of D'.

Additional simulation methods

We report our simulations using the ADEMP (aims, data-generating mechanisms, estimands, methods, and performance measures) approach:[10]

Aims: The aim of this simulation was to explore if Two-step *cis*-MR can be used attenuate bias in a *cis*-MR analysis when there are multiple known biasing pathways. We explore two settings: 1) when the two pathways are independent, and 2) when they are not independent. For simplicity, in the second setting, we simulated a setting in which two variables on the same pathway were adjusted for.

Data-generating mechanisms: The directed acyclic graph (DAG) in Supplementary Figures 1 and 2 were used as the basis for the data generation mechanism for the simulation. As before, this would simulate a setting in which the bias occurs through a pleiotropic SNP or a confounding by a SNP in perfect LD. However, unlike in the previous setting, both data generative models included two variables for adjustment. All adjusted variables were simulated as random normal variables with a mean of zero and standard deviation of 1. Again, their causal effects were simulated as 1. The parameters were otherwise the same as in the previous simulation. The simulations were again repeated 100,000 times.

Estimands and other targets: The estimand was the average causal effect of intervening and changing the exposure of each individual from its observed level x by a single unit, or E[Y(X=x)] – E[Y(X=x-1)] using potential outcomes framework.

Methods: We compare two methods of estimating the variant-outcome association: 1) the crude association that would be derived from a GWAS, 2) the adjusted association that would be derived by using Two-step *cis*-MR. The confounder-outcome association was estimated as the Wald ratio of association the non-*cis* genetic risk score for the confounder (GRS) with the outcome divided by the GRS-confounder association. The standard error was estimated as the standard error of the confounder (GRS) with the outcome divided by the absolute value of the GRS-confounder association.

Performance measure: The performance measure was the estimate of the casual effect, and the standard error in the estimate of the causal effect for the crude and Two-step *cis*-MR methods in settings in which multiple biasing pathways are adjusted for.

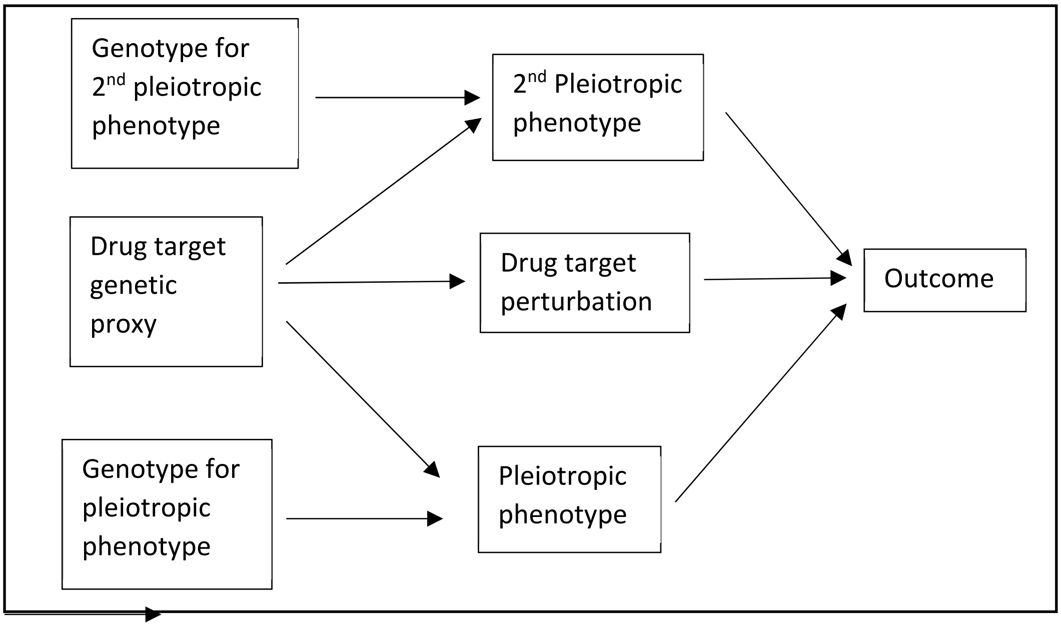

Figure S1: Directed Acyclic Graph (DAG) representing bias through pleiotropy or two variants in perfect LD, when there are two independent variables for adjustment.

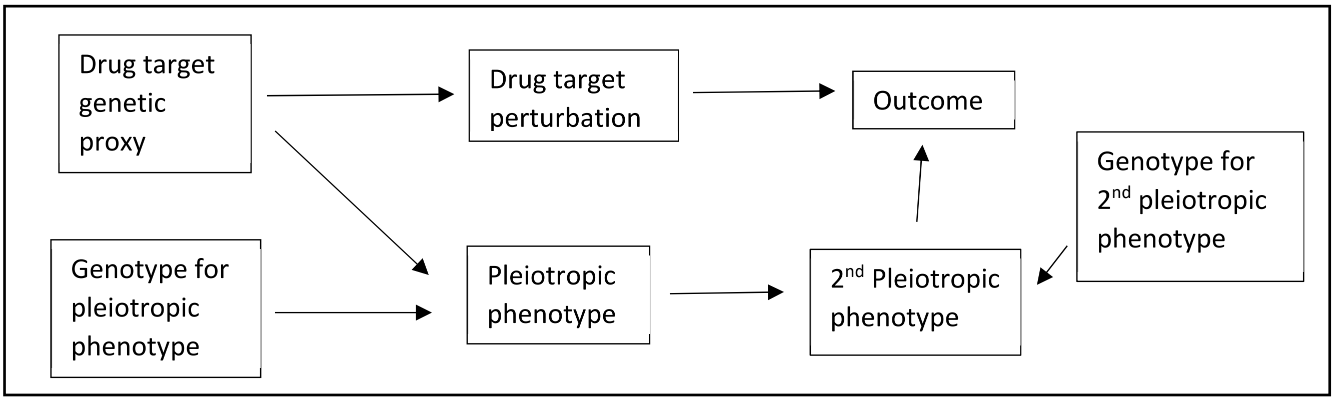

Figure S2: Directed Acyclic Graph (DAG) representing bias through pleiotropy or two variants in perfect LD, when there are two non-independent variables for adjustment.

References

10. Morris TP, White IR, Crowther MJ. Using simulation studies to evaluate statistical methods. Statistics in Medicine. 2019;38(11):2074–102.
